# Association of water intake and hydration status with risk of kidney stone formation based on NHANES 2009–2012 cycles

**DOI:** 10.1017/S1368980022001033

**Published:** 2022-09

**Authors:** Jie-Sian Wang, Hsiu-Yin Chiang, Hung-Lin Chen, Martha Flores, Ana Navas-Acien, Chin-Chi Kuo

**Affiliations:** 1 Division of Nephrology, Department of Internal Medicine, China Medical University Hospital, College of Medicine, China Medical University, 2, Yude Road, North District, Taichung, 404, Taiwan; 2 Graduate Institute of Biomedical Sciences, China Medical University, Taichung, Taiwan; 3 Big Data Center, China Medical University Hospital, China Medical University, Taichung, Taiwan; 4 US Department of Health and Human Services, Health Resources and Services Administration, Rockville, MD, USA; 5 Department of Environmental Health Sciences, Mailman School of Public Heath, Columbia University, New York, NY, USA

**Keywords:** Water intake, Fluid intake, Hydration status, Kidney stone, Nephrolithiasis, Urine output

## Abstract

**Objective:**

Evaluating the association of water intake and hydration status with nephrolithiasis risk at the population level.

**Design:**

It is a cross-sectional study in which daily total plain water intake and total fluid intake were estimated together with blood osmolality, urine creatinine, urine osmolality, urine flow rate (UFR), free water clearance (FWC) and urine/blood osmolality ratio (U_osm_:B_osm_). The associations of fluid intake and hydration markers with nephrolithiasis were evaluated using multivariable logistic regression.

**Setting:**

General US population.

**Participants:**

A total of 8195 adults aged 20 years or older from the National Health and Nutritional Examination Survey 2009–2012 cycles.

**Results:**

The population medians (interquartile ranges, IQR) for daily total plain water intake and total fluid intake were 807 (336–1481) and 2761 (2107–3577) ml/d, respectively. The adjusted OR (95 % CI) of nephrolithiasis for each IQR increase in total plain water intake and total fluid intake were 0·92 (95 % CI 0·79, 1·06) and 0·84 (95 % CI 0·72, 0·97), respectively. The corresponding OR of nephrolithiasis for UFR, blood osmolality, U_osm_:B_osm_ and urine creatinine were 0·87 (95 % CI 0·76, 0·99), 1·18 (95 % CI 1·06, 1·32), 1·38 (95 % CI 1·17, 1·63) and 1·27 (95 % CI 1·11, 1·45), respectively. A linear protective relationship of fluid intake, UFR and FWC with nephrolithiasis risk was observed. Similarly, positive dose–response associations of nephrolithiasis risk with markers of insufficient hydration were identified. Encouraging a daily water intake of >2500 ml/d and maintaining a urine output of 2 l/d was associated with a lower prevalence of nephrolithiasis.

**Conclusion:**

This study verified the beneficial role of general water intake recommendations in nephrolithiasis prevention in the general US population.

The incidence and prevalence of kidney stone formation (i.e. nephrolithiasis) are increasing globally^(1,2)^. Nephrolithiasis is linked to several chronic illnesses including CVD, obesity and diabetes^(3–6)^. Although increasing fluid intake is proposed as an essential factor for preventing kidney stones and is extensively implemented in daily practice^(4,7–9)^, few studies have explicitly quantified nephrolithiasis risk based on objective measurements of hydration status at the population level.

Accurately quantifying hydration status is a major challenge in epidemiological research. Although many hydration indicators have been proposed, technical requirements increase when more precise measurement methods for hydration status are required, such as isotope dilution or bioimpedance^(10)^. However, at the population level, more cost-effective methods such as urine osmolality, urine/blood osmolality ratio (U_osm_:B_osm_) and free water clearance (FWC) may be preferred; these methods have been used in clinical medicine studies to evaluate Na and water homoeostasis^(11–14)^.

The National Health and Nutritional Examination Survey (NHANES) 2009–2012 cycles provide notable information on water and fluid intake and hydration status indicators such as urine osmolality and urine excretion rate. We used these measurements to investigate the association of water and fluid intake and hydration status with nephrolithiasis risk in a representative sample of US adults. We hypothesised that insufficient water or fluid intake and the resulting dehydration is associated with a higher risk of nephrolithiasis.

## Materials and methods

### Study population

NHANES 2009–2012 was conducted by the US National Center for Health Statistics by using a stratified multistage sampling design to obtain a nationally representative sample of the civilian non-institutionalised population of the USA. A total of 11 778 adults aged 20 years or older completed the NHANES 2009–2012 in-home interview and medical evaluation at mobile examination centres (MEC). The participation rates for interviews and examinations among participants aged 20 years and older were 71·4 % and 69·2 %, respectively. Of the 10 125 participants whose urine osmolality and UFR were measured, we excluded 604 who lacked blood osmolality, creatinine and urine creatinine data, 95 who were pregnant, 940 who lacked alcohol consumption data, and 291 who lacked other covariate data (BMI, education level, and the status of diabetes and hypertension). The final sample size for the present study was 8195.

### Measurement of daily water and fluid intake and hydration indicators

The NHANES 2009–2012 cycles included two 24-h dietary recalls for 87·5 % of the study participants, who were asked to list the types and amount food and beverages that they consumed to produce data that is representative of their usual dietary intake^(15)^. The first dietary interview was conducted in-person during the MEC assessment to record the dietary intake for the previous 24 h and the second-day dietary interview was performed through a phone follow-up 3–10 d later^(16)^. Total plain water intake (g/d) was defined as the volume of water consumed (including plain tap water, water from drinking fountains or water coolers, bottled water, and spring water) over 24 h^(17)^. Total fluid intake (g/d) was defined as the daily aggregate of water (moisture) intake, which consisted of all water present in foods and beverages (including tap and bottled water consumed as beverages) and was calculated using the US Department of Agriculture’s Food and Nutrient Database for Dietary Studies 5 (FNDDS 5·0)^(17,18)^. We assumed the 2-d mean to be a practical estimation of the average intake of each participant and used it for statistical analyses.

While there is no gold standard marker of hydration status, we proposed several hydration indicators based on current knowledge and existing literature^(19–21)^. Adjusting for urine osmolality or urine creatinine is a common approach to correct over-concentrated or over-diluted urine in human biomonitoring for environmental exposures, such as arsenic^(21,22)^. Urine output is a marker for hydration status and varies inversely with the body hydration status; however, there is no consensus on the cut-off of urine volume to define hydration status^(23,24)^. On the other hand, FWC is a physiological marker of body water balance and U_osm_:B_osm_ has been found closely related to FWC^(14,25)^. Therefore, both FWC and U_osm_:B_osm_ were selected as the indicators of hydration in the present study. These indicators are described as follows:

### Urine osmolality

In NHANES 2009–2012, urine samples were directly analysed at MEC within 4 h of collection^(26)^. Urine osmolality was measured using the Osmette II Model 5005 Automatic Osmometer (Precision Systems Inc.) and the freezing point depression method. Osmolality is expressed as milliosmoles (mOsm) per kilogram of water.

### Urine creatinine

Urine creatinine is commonly used to correct other urine markers for urine dilution in spot urine samples in both clinical and epidemiological research^(27)^. It was measured using an enzymatic (creatinase) method during the 2009–2012 cycles.

### Urine output

In NHANES 2009–2012, the time between two urinations was recorded for each participant, and this procedure was repeated until the requested volume of urine was collected^(28)^. For the first void, participants were asked to record their time of last void before the MEC visit and were then asked to void at MEC where the time of urination and volume of the urine were recorded^(29)^. If the urine volume was insufficient, participants were asked for the second or third voids at MEC^(29)^. A maximum of three urine samples were collected from each participant. All urine samples with insufficient volume were refrigerated. Additional urine samples were then pooled and mixed with the previous samples before undergoing specimen processing. Urine flow rate (UFR) was obtained by dividing the total collection time by total volume collected at MEC. UFR is standardised to body weight and converted into the conventional unit for expressing urine output (ml/kg/h).

### Free water clearance

FWC (C_H2O_) is the volume of solute-free water in urine that has been eliminated from plasma/min. FWC can be positive or negative (–1 to 0 ml/min is considered normal) and used as an indicator of hydration status; it calculated as follows^(11,30)^:

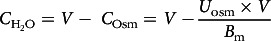

where *C*_osm_ is the osmolar clearance, *U*_osm_ is the urine osmolality (mOsm/kg), *B*_osm_ is the blood osmolality (mOsm/kg) and *V* is the UFR (ml/kg/h). In the present study, a unit of FWC corresponded to UFR (ml/kg/h).

### Urine/blood osmolality ratio

Although FWC is widely used as a diagnostic tool for Na disorder and a theoretical benchmark for evaluating hydration status^(31)^, implementing it in epidemiological research is difficult because three parameters are required. Urine–blood osmolality estimation, which is easier to implement, was demonstrated to approximate FWC estimations (*r* ^*2*^ = 0·86)^(14)^.

### Outcome measurement

The primary outcome was the response to the question, ‘Have you ever had kidney stones?’ Participants who responded ‘yes’ were defined as kidney stone formers. The secondary outcome was based on participants’ response to the question, ‘How many times have you passed a kidney stone?’ We considered any participant who reported passing at least two stones as a recurrent stone former; those who had passed only one stone were considered first stone formers.

### Other variables

Sociodemographic variables collected during the interview included age, race/ethnicity, sex, education, smoking status and alcohol consumption status. Smoking status was self-reported with participants being categorised as current smokers, former smokers or never smokers. Alcohol consumption was categorised as never (< 12 drinks in any 1 year in life), former (≥ 12 drinks in any 1 year in life and not drinking now) and current (≥12 drinks in any 1 year in life and drinking now) drinking^(32)^.

BMI was calculated by dividing measured weight (kg) by measured height squared (m^2^) and classified as normal or underweight (BMI < 25 kg/m^2^), overweight (BMI 25–30 kg/m^2^) or having obesity (BMI > 30 kg/m^2^). Hypertension was defined as a self-reported physician diagnosis (whether participants were ever told to have hypertension by a health professional) characterised by the use of antihypertensive medication and a mean systolic blood pressure > 140 mmHg or mean diastolic blood pressure > 90 mmHg. Diabetes mellitus was defined as a self-reported physician diagnosis characterised by medication use or a glucose level of ≥126 mg/dl with ≥8 h of fasting or ≥200 mg/dl with <8 h of fasting. Chronic kidney disease was defined as having an estimated glomerular filtration rate of < 60 ml/min/1·73 m^2^. The use of diuretics may directly influence hydration status and be a potential confounding factor; therefore, we specifically collected information on diuretic use to evaluate its effect on the hypothesised exposure–outcome relationship. Information on prescription medication use was collected through household interviews conducted by trained interviewers, who used a computer-assisted personal interviewing system to record the specific product name indicated on the medication label^(18)^.

### Statistical analysis

Statistical analyses were performed using the sample survey commands found in STATA version 12.0 (StataCorp. LP) to account for the complex sampling design of NHANES 2009–2012 and obtain unbiased point estimates. The two-sided statistical significance level was *α* = 0·05. Descriptive statistics were computed as unadjusted means and standard deviations and compared using Student’s *t* test for continuous variables and a chi-squared test for categorical variables.

Multivariable logistic regression was used to verify the association of kidney stone formation with water and fluid intake and hydration status indicators. The models were initially adjusted for sociodemographic and lifestyle variables including age, sex, race/ethnicity (non-Hispanic White/non-Hispanic Black/Mexican-American/Others) and education (less than high school/high school/higher than high school) followed by BMI, smoking status (never/former/current), alcohol consumption (never/former/current) and comorbidities including hypertension, diabetes mellitus, estimated glomerular filtration rate, and the use of diuretics. We also conducted a multiple logistic regression of the full model for each dietary variable and hydration indicator restricted to participants with normal kidney function defined by an estimated glomerular filtration rate of greater than 60 ml/min/1·73 m^2^. The volume of daily water intake and daily fluid intake were right-skewed and log-transformed to improve normality. Independent variables comprising total daily water intake, total fluid intake and all the hydration status indicators were entered into separate regression models as quartiles and continuous variables to obtain effect sizes per interquartile range (IQR) and with restricted quadratic splines. With the aforementioned adjustment strategy, multinomial logistic analyses were performed to examine the association between hydration status-related factors and the likelihood of being a first or recurrent kidney stone former.

## Results

Participants with a history of nephrolithiasis tended to be older, and an upward trend was observed in the proportion of first and recurrent kidney formers who were male and non-Hispanic White and had obesity (Table 1). Chronic non-communicable diseases such as hypertension, diabetes, chronic kidney disease, albuminuria and hyperuricemia were more prevalent among stone formers (Table 1). Daily water and fluid intake were not significantly different among participants with and without nephrolithiasis, although stone formers tended to report less plain water intake (Table 1). The proportion of participants who reported not drinking any plain water in a day (12·4 %) was higher for participants with recurrent nephrolithiasis relative to the other participants, but this difference was not significant compared with stone-free participants and first stone formers. Excluding FWC, all other hydration status indicators indicated that stone formers were relatively dehydrated compared with stone-free participants (Table 1).

**Table 1:** Demographics of participants without kidney stone *v*. first kidney stone *v*. recurrent kidney stone

Characteristics	All participants		Participants without kidney stone		Participants with first kidney stone		Participants with recurrent stone		*P*-value
	*n*	%	*n*	%	*n*	%	*n*	%	
*n*	8195		7464		511		220		
Age	47·5	0·51	46·9	0·56	54·1	0·65	53·1	1·05	<0·01
Sex (male)	50·5	0·6	50·1	0·6	53·3	2·7	57·7	4·0	0·12
Race									<0·01
Non-Hispanic White	70·2	2·4	69·5	2·4	74·2	2·8	84·0	3·8	
Non-Hisptanic Black)	10·2	1·1	10·6	1·2	6·8	1·3	2·3	0·7	
Mexican-American	7·5	1·3	7·7	1·3	7·0	1·9	4·1	1·3	
Others	12·2	1·1	12·3	1·1	12·0	1·9	9·7	3·2	
Education									0·15
< High school	5·4	0·4	5·3	0·4	7·4	1·2	4·1	0·9	
= High school	32·4	1·4	32·0	1·3	35·8	3·2	38·7	6·4	
>High school	62·2	1·5	62·7	1·5	56·8	3·1	57·2	6·4	
Smoking									0·18
Never	55·1	1·2	55·4	1·1	53·7	4·0	46·4	5·0	
Former	25·1	1·0	24·8	1·0	28·8	2·8	26·8	4·4	
Current	19·9	0·8	19·8	0·8	17·5	3·1	26·7	2·6	
Alcohol									0·39
Never	26·0	0·8	25·7	0·9	28·3	2·0	29·4	5·3	
Former	31·9	0·9	32·3	1·0	26·8	3·0	29·8	3·1	
Current	42·1	1·0	42·0	1·0	44·9	3·1	40·8	5·0	
BMI									<0·01
<25 kg/m^2^	30·3	1·2	31·4	1·2	21·3	2·6	15·4	2·9	
25–30 kg/m²	34·4	0·9	34·3	1·0	36·1	3·0	33·5	3·2	
>30 kg/m²	35·3	0·9	34·3	0·9	42·6	2·6	51·1	3·5	
Hypertension	38·8	0·9	37·5	1·0	53·7	3·0	49·2	4·5	<0·01
Diabetes mellitus	10·1	0·4	9·2	0·5	19·1	2·1	19·6	3·1	<0·01
CKD*	6·4	0·4	6·0	0·4	12·5	1·5	7·6	2·0	<0·01
Albuminuria	7·5	0·4	7·2	0·4	11·8	1·8	8·8	2·1	0·03
Hyperuricemia	17·4	0·7	17·0	0·7	21·8	2·2	19·9	3·3	0·02
Hyperlipidemia	43·0	0·9	43·3	1·0	39·6	2·8	42·2	3·6	0·41

	Median	IQR	Median	IQR	Median	IQR	Median	IQR	
Total fluid intake (ml/d) (*n* 7169)	2761	2107–3577	2772	2113–3601	2616	1906–3414	2760	2225–3370	0·12
Daily plain water intake (ml/d)	807	336–1481	814	355–1500	770	259–1406	748	222–1407	0·05

	*n*	%	*n*	%	*n*	%	*n*	%	
No water intake	9·4	0·6	9·4	0·5	8·4	1·5	12·4	2·8	0·46

	Median	IQR	Median	IQR	Median	IQR	Median	IQR	
Urine creatinine (mg/dl)	105	58–162	103	56–162	123	73–162	110	80–166	0·01
Blood osmolality (mOsm/kg)	278	275–281	278	275–281	279	276–282	278	276–282	<0·01
Urine osmolality (mOsm/kg)	634	385–820	631	378–821	656	462–809	684	467–808	<0·01
Urine/blood osmolality ratio	2·3	1·4–2·9	2·3	1·4–3·0	2·4	1·7–2·9	2·5	1·7–2·9	<0·01
Urine flow rate (ml/kg/h)	0·6	0·4–1·1	0·6	0·4–1·1	0·5	0·3–0·9	0·5	0·3–0·8	<0·01
Free water clearance (ml/kg/h)	−0·6	-1·0–0·2	−0·6	-1·0–0·2	−0·6	-0·9–0·3	−0·6	-0·9–-0·3	0·32

	*n*	%	*n*	%	*n*	%	*n*	%	
Diuretics use	11·9	0·6	11·2	0·6	18·0	1·8	22·4	3·0	<0·01

CKD, chronic kidney disease.

* CKD is defined as estimated glomerular filtration rate less than 60 ml/min/1·73 m^2^.

Continuous variables were expressed as medians and interquartile ranges, and categorical variables were expressed as frequency (percentage).

The medians and IQR of daily water and fluid intake and hydration status indicators are summarised in Supplemental Figs. 1–3. The population medians (IQR) for daily total plain water intake and total fluid intake were 807 (336–1481) and 2761 (2107–3577) ml/d, respectively. Total daily water and fluid intake progressively decreased with age because elder participants tended to produce less concentrated urine and were more hydrated relative to younger participants (see online Supplemental Figs. 1–3). Compared with women, men reported higher daily water and fluid intake, more concentrated urine, and stronger intentions to drink water. However, urine output was comparable between the male and female participants (0·63 *v*. 0·65 ml/kg/h). Although non-Hispanic White participants tended to report higher fluid intake and more diluted urine than non-Hispanic Black participants did, FWC levels were comparable between these two ethnic groups. Participants with overweight or obese reported higher fluid intake, more concentrated urine and less urine output relative to participants with normal weight (see online Supplemental Figs. 1 and 2). Comorbidities such as hypertension and chronic kidney disease were related to less daily fluid intake but were also associated with more diluted urine (see online Supplemental Figs. 1 and 2). Spearman’s correlation coefficients for all analysed independent factors are summarised in Supplemental Fig. 4.

**Fig. 1:**
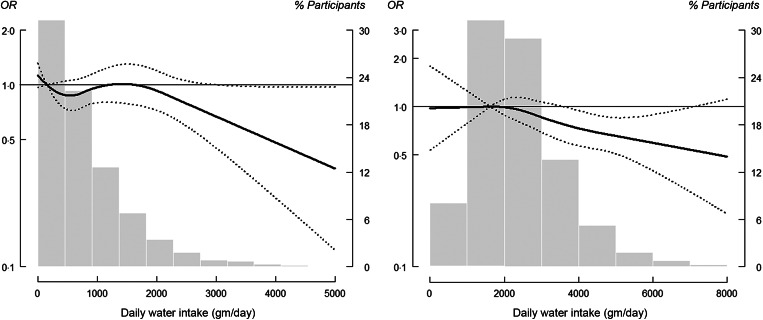
OR of kidney stone formation (determined by water and fluid intake measurements). Solid lines represent adjusted OR based on restricted quadratic splines for daily water and fluid intake measurements with knots at the 10th, 50th and 90th percentiles (corresponding to 39·5, 807·2 and 2281·1 g/d, respectively, for daily water intake; and 1627·6, 2761·6 and 4613·6 g/d, respectively, for daily fluid intake). Dotted lines represent 95 % CI. The reference was set at the 10th percentile of the distribution. Adjustment factors were identical to those used in Model 3 (Table 2). Bars represent a histogram of distribution of daily water and fluid measurements among participants (extreme tails of the histogram were truncated)

**Fig. 2:**
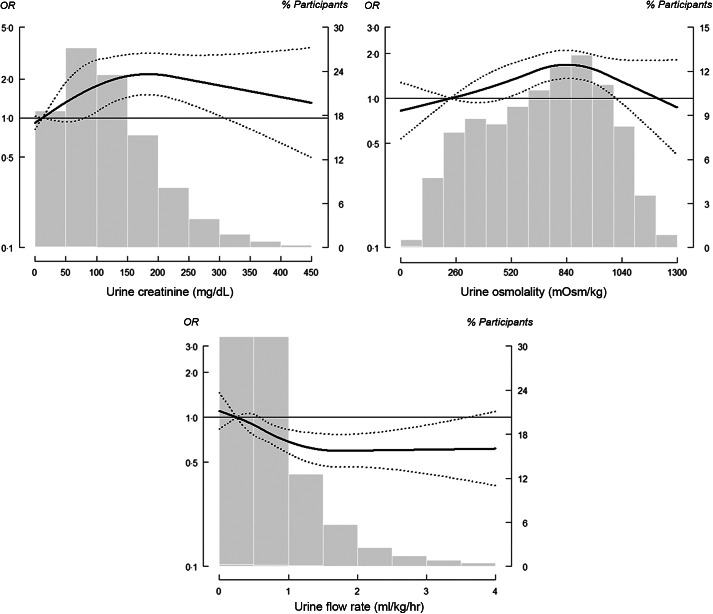
OR of kidney stone formation (determined by common urine concentration indicators). Solid lines represent adjusted OR based on restricted quadratic splines for common urine concentration indicators (including urine creatinine, osmolality and flow rate) with knots at the 10th, 50th and 90th percentiles (corresponding to 33, 105 and 220 mg/dl, respectively, for urine creatinine; 233, 634 and 820 mOsm/kg, respectively, for urine osmolality; and 0·25, 0·62 and 1·75 ml/kg/h, respectively, for urine flow rate). Dotted lines represent 95 % CI. Reference was set at the 10th percentile of the distribution. Adjustment factors were identical to those used in Model 3 (Table 2). Bars represent a histogram of the distribution of analysed urine concentration indicators among participants (extreme tails of the histogram were truncated)

The adjusted OR (95 % CI) of nephrolithiasis comparing the IQR for total plain water intake and total fluid intake were 0·92 (95 % CI 0·79, 1·06) and 0·84 (95 % CI 0·72, 0·97), respectively (Table 2, Model 3). However, a non-linear inverse association at higher levels of water and fluid intake was observed, which indicated a threshold dose–response relationship (Fig. 1). The corresponding OR of having a history of kidney stones for each incremental IQR increase in urine creatinine, urine osmolality and UFR were 1·27 (95 % CI 1·11, 1·45), 1·39 (95 % CI 1·18, 1·64) and 0·87 (95 % CI 0·76, 0·99), respectively (Table 2, Model 3). The dose–response relationship for both urine creatinine and urine osmolality plateaued with no longer significant increases after urine creatinine reached 300 mg/dl and urine osmolality reached 1000 mOsm/kg (Fig. 2). For urine output, a non-linear inverse response that plateaued at 1·5 ml/kg/h was observed (Fig. 2).

**Table 2:** OR (95 % CI) of having a history of kidney stones by total plain water intake, total fluid intake and multiple hydration indicators in quartiles

	N(C/NC)	Model 1		Model 2		Model 3		Model 4*	
		OR	95 % CI	OR	95 % CI	OR	95 % CI	OR	95 % CI
Dietary variables									
Total plain water intake (ml/d)									
Quartile 1 (≤ 451·5)	185/1545	1·00		1·00		1·00		1·00	
Quartile 2 (>451·5 and ≤ 859·1)	135/1579	0·66	0·50, 0·89	0·72	0·53, 0·96	0·71	0·53, 0·97	0·67	0·49, 0·91
Quartile 3 (>859·1 and ≤ 1477·6)	150/1443	0·93	0·64, 1·34	1·09	0·77, 1·55	1·06	0·73, 1·55	1·06	0·71, 1·57
Quartile 4 (>1477·6)	124/1325	0·71	0·50, 0·99	0·89	0·63, 1·26	0·82	0·58, 1·15	0·81	0·57, 1·16
*P*-trend		0·15		0·90		0·59		0·63	
Per log-transformed IQR		0·85	0·73, 0·98	0·94	0·81, 1·09	0·92	0·79, 1·06	0·91	0·77, 1·08
Total fluid intake (ml/d)									
Quartile 1 (≤ 2070·3)	225/2030	1·00		1·00		1·00		1·00	
Quartile 2 (>2070·3 and ≤ 2705·3)	179/1659	0·92	0·67, 1·28	0·92	0·65, 1·30	0·88	0·63, 1·23	0·87	0·59, 1·27
Quartile 3 (>2705·3 and ≤ 3534·5)	146/1463	0·98	0·73, 1·32	0·98	0·73, 1·32	0·92	0·69, 1·23	0·94	0·69, 1·29
Quartile 4 (>3534·5)	109/1360	0·70	0·50, 0·98	0·73	0·50, 1·06	0·67	0·46, 0·97	0·64	0·43, 0·94
*P*-trend		0·05		0·12		0·04		0·03	
Per log-transformed IQR		0·86	0·76, 0·98	0·87	0·75, 1·01	0·84	0·72, 0·97	0·83	0·71, 0·97
Hydration indicators									
Urine flow rate (ml/kg/h)									
Quartile 1 (≤ 0·38)	241/2046	1·00		1·00		1·00		1·00	
Quartile 2 (>0·38 & ≤ 0·62)	201/1890	0·92	0·73, 1·16	0·92	0·72, 1·18	0·98	0·76, 1·27	0·99	0·75, 1·30
Quartile 3 (>0·62 & ≤ 1·06)	164/1817	0·64	0·54, 0·77	0·65	0·53, 0·78	0·73	0·59, 0·90	0·73	0·57, 0·93
Quartile 4 (>1·06)	125/1713	0·54	0·42, 0·69	0·56	0·44, 0·72	0·67	0·52, 0·87	0·63	0·47, 0·85
*P*-trend		<0·01		<0·01		<0·01		<0·01	
Per IQR		0·79	0·70, 0·90	0·81	0·71, 0·92	0·87	0·76, 0·99	0·84	0·72, 0·98
Free water clearance (ml/kg/h)									
Quartile 1 (≤ -0·98)	157/1807	1·00		1·00		1·00		1·00	
Quartile 2 (>-0·98 & ≤ -0·59)	193/1813	1·39	0·97, 1·98	1·23	0·86, 1·75	1·18	0·83, 1·66	1·20	0·83, 1·73
Quartile 3 (>-0·59 & ≤ -0·23)	227/2027	1·45	1·06, 1·97	1·14	0·84, 1·57	1·03	0·75, 1·41	1·10	0·80, 1·52
Quartile 4 (>-0·23)	154/1819	0·98	0·67, 1·43	0·82	0·56, 1·21	0·79	0·54, 1·16	0·74	0·48, 1·13
*P*-trend		0·91		0·24		0·15		0·14	
Per IQR		0·98	0·91, 1·05	0·93	0·85, 1·01	0·92	0·84, 1·01	0·91	0·83, 1·00
Urine osmolality (mOsm/kg)									
Quartile 1 (≤ 379)	122/1771	1·00		1·00		1·00		1·00	
Quartile 2 (>379 and ≤ 622)	218/1915	1·82	1·37, 2·42	1·61	1·23, 2·12	1·48	1·12, 1·95	1·57	1·18, 2·09
Quartile 3 (>622 and ≤ 817)	231/1836	1·98	1·59, 2·47	1·97	1·57, 2·46	1·80	1·44, 2·23	1·87	1·48, 2·37
Quartile 4 (>817)	160/1944	1·36	1·07, 1·74	1·69	1·33, 2·15	1·57	1·20, 2·04	1·63	1·21, 2·18
*P*-trend		<0·01		<0·01		<0·01		<0·01	
Per IQR		1·21	1·06, 1·39	1·45	1·24, 1·68	1·39	1·18, 1·64	1·42	1·18, 1·70
Blood osmolality (mOsm/kg)									
Quartile 1 (≤ 275)	147/2095	1·00		1·00		1·00		1·00	
Quartile 2 (>275 and ≤ 278)	169/1947	1·29	0·96, 1·74	1·19	0·87, 1·62	1·17	0·86, 1·59	1·15	0·84, 1·55
Quartile 3 (>278 and ≤ 281)	175/1768	1·28	0·98, 1·67	1·08	0·85, 1·39	1·06	0·82, 1·35	1·06	0·83, 1·36
Quartile 4 (>281)	240/1656	1·91	1·45, 2·52	1·35	1·05, 1·72	1·20	0·91, 1·59	1·28	0·97, 1·68
*P*-trend		<0·01		0·05		0·30		0·14	
Per IQR		1·44	1·28, 1·62	1·24	1·13, 1·37	1·18	1·06, 1·32	1·22	1·07, 1·39
Urine/blood osmolality ratio								*n* 3997	
Quartile 1 (≤ 1·35)	125/1764	1·00		1·00		1·00		1·00	
Quartile 2 (>1·35 and ≤ 2·23)	214/1932	1·74	1·32, 2·29	1·55	1·18, 2·03	1·42	1·09, 1·85	1·55	1·16, 2·05
Quartile 3 (>2·23 and ≤ 2·92)	235/1824	1·99	1·61, 2·44	1·97	1·58, 2·46	1·82	1·46, 2·26	1·94	1·52, 2·46
Quartile 4 (>2·92)	157/1946	1·31	1·02, 1·68	1·64	1·28, 2·10	1·53	1·17, 2·01	1·62	1·20, 2·18
*P*-trend		0·01		<0·01		<0·01		<0·01	
Per IQR		1·19	1·04, 1·36	1·43	1·23, 1·67	1·38	1·17, 1·63	1·40	1·17, 1·69
Urine creatinine (mg/dl)									
Quartile 1 (≤ 61)	128/1733	1·00		1·00		1·00		1·00	
Quartile 2 (> 61 and ≤ 105)	199/1901	1·61	1·11, 2·34	1·61	1·11, 2·33	1·49	1·02, 2·15	1·53	1·08, 2·16
Quartile 3 (> 105 and ≤ 161)	220/1831	1·92	1·51, 2·46	2·01	1·55, 2·61	1·85	1·42, 2·40	1·97	1·51, 2·56
Quartile 4 (> 161)	184/2001	1·58	1·17, 2·13	1·95	1·40, 2·70	1·79	1·26, 2·54	1·90	1·33, 2·70
*P*-trend		<0·01		<0·01		<0·01		0·02	
Per IQR		1·14	1·03, 1·26	1·32	1·16, 1·49	1·27	1·11, 1·45	1·33	1·15, 1·53

IQR, interquartile range; eGFR, estimated glomerular filtration rate.

* After restricting to participants with normal kidney function, the numbers of participants for total plain water/fluid intake and other hydration indicators were 3103, 3491 and 3977, respectively.

Model 1: univariate analysis.

Model 2: further adjusted for age, sex, race/ethnicity and education levels.

Model 3: further adjusted for BMI, smoking, alcohol, hypertension, diabetes mellitus, eGFR and diuretics use.

Model 4: further restricted to participants with normal kidney function (eGFR≥ 60 ml/min/1·73 m^2^).

**Fig. 3:**
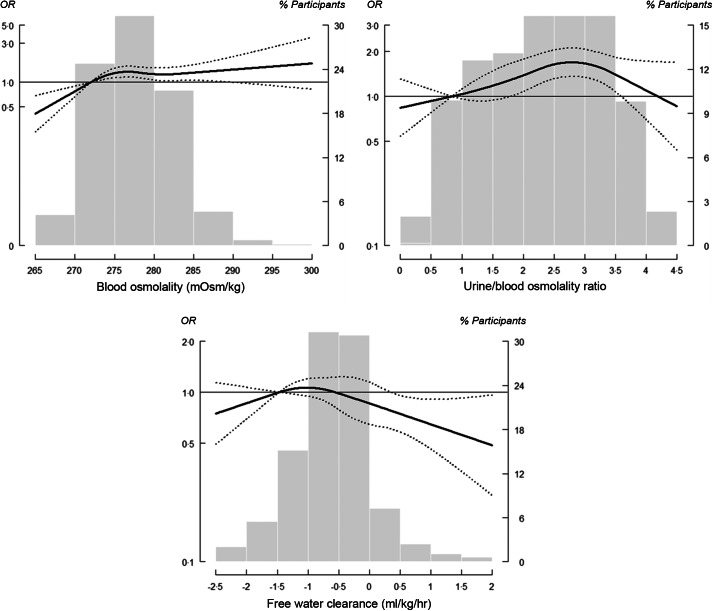
OR of kidney stone formation (determined by blood osmolality-based hydration indices). Solid lines represent adjusted OR based on restricted quadratic splines for blood osmolality-based hydration indices (including blood osmolality, osmolality ratio of urine to blood and free water clearance (FWC) with knots at the 10th, 50th and 90th percentiles (corresponding to 272, 278 and 283 mg/dl, respectively, for urine creatinine; and 0·84, 2·28 and 3·44 mOsm/kg, respectively, for urine osmolality; and –1·47, –0·59 and 0·2 ml/kg/h, respectively, for FWC). Dotted lines represent upper and lower 95 % CI. Reference was set at the 10th percentile of the distribution. Adjustment factors were identical to those used in Model 3 (Table 2). Bars represent a histogram of distribution of analysed urine concentration indicators among participants (extreme tails of the histogram were truncated)

For blood osmolality-based indicators of hydration status, the fully adjusted OR (95 % CI) of nephrolithiasis for comparing the highest and lowest quartiles of blood osmolality, U_osm_:B_osm_ and FWC were 1·20 (95 % CI (0·91, 1·59); *P*-value for trend = 0·30), 1·53 (95 % CI (1·17, 2·01), *P*-value for trend < 0·01) and 0·79 (95 % CI (0·54, 1·16); *P*-value for trend = 0·15), respectively (Table 2, Model 3). The dose–response relationship of nephrolithiasis and blood osmolality was curvilinear up to 283 mOsm/kg and thereafter exhibited a shallow linear slope (Fig. 3). The dose–response curve of U_osm_:B_osm_ was similar to that of urine osmolality. For FWC, a significant and extreme protective dose–response was observed beyond 0·5 ml/kg/h. In our sensitivity analysis, the statistical inference remained consistent even after we restricted our sample to participants with normal kidney function, which was defined as an estimated glomerular filtration rate of  > 60 ml/min/1·73 m^2^ (Table 2, Model 4).

Our multinomial logistic regression analysis revealed differential associations between the risk of first and recurrent kidney stone formation, and indicators of hydration status were identified for urine output and FWC. The fully adjusted OR (95 % CI) of having first and recurrent nephrolithiasis were 0·85 (95 % CI (0·65, 1·12)) and 0·70 (95 % CI (0·49, 0·99)) for each incremental IQR increase in urine output, respectively; for FWC, the corresponding OR were 0·96 (95 % CI (0·87, 1·06)) and 0·86 (95 % CI (0·75, 0·99)), respectively (Table 3).

**Table 3:** OR (95 % CI) of first and recurrent stone formation by total plain water intake, total fluid intake and multiple hydration indicators per the log-transformed interquartile range. Reference group comprises stone-free participants

Hydration indicators	*n*	Model 1		Model 2		Model 3		Model 4†	
		OR	95 % CI	OR	95 % CI	OR	95 % CI	OR	95 % CI
Dietary variables									
Total plain water intake (ml/d)*	5931								
First stone former		0·83	0·70, 0·98	0·92	0·78, 1·10	0·90	0·76, 1·08	0·92	0·74, 1·14
Recurrent stone former		0·89	0·68, 1·16	0·98	0·72, 1·32	0·94	0·70, 1·26	0·89	0·67, 1·20
Total fluid intake (g/d)*	7171								
First stone former		0·80	0·68, 0·94	0·83	0·69, 0·99	0·81	0·67, 0·97	0·82	0·67, 1·01
Recurrent stone former		1·00	0·83, 1·21	0·96	0·73, 1·25	0·89	0·69, 1·14	0·84	0·65, 1·09
Hydration indicators									
Urine flow rate (ml/kg/h)	8187								
First stone former		0·76	0·57, 1·00	0·79	0·60, 1·04	0·85	0·65, 1·12	0·81	0·60, 1·08
Recurrent stone former		0·60	0·40, 0·90	0·60	0·40, 0·89	0·70	0·49, 0·99	0·68	0·48, 0·96
Free water clearance (ml/kg/h)	8187								
First stone former		1·00	0·93, 1·09	0·96	0·87, 1·05	0·96	0·87, 1·06	0·94	0·85, 1·03
Recurrent stone former		0·93	0·84, 1·03	0·88	0·78, 0·99	0·86	0·75, 0·99	0·86	0·75, 0·99
Urine osmolality (mOsm/kg)	8195								
First stone former		1·19	0·96, 1·48	1·43	1·09, 1·86	1·38	1·04, 1·82	1·42	1·07, 1·90
Recurrent stone former		1·25	1·00, 1·57	1·49	1·11, 2·00	1·42	1·03, 1·96	1·41	1·02, 1·95
Blood osmolality (mOsm/kg**)**	8195								
First stone former		1·47	1·27, 1·71	1·26	1·11, 1·43	1·21	1·04, 1·40	1·23	1·03, 1·46
Recurrent stone former		1·37	1·21, 1·54	1·21	1·09, 1·34	1·14	1·01, 1·28	1·21	1·07, 1·36
Urine/blood osmolality ratio	8195								
First stone former		1·17	0·94, 1·44	1·41	1·08, 1·84	1·37	1·03, 1·81	1·41	1·06, 1·88
Recurrent stone former		1·23	0·98, 1·55	1·48	1·10, 1·99	1·41	1·02, 1·96	1·40	1·01, 1·94
Urine creatinine (mg/dl)	4366								
First stone former		1·15	0·99, 1·33	1·32	1·11, 1·58	1·29	1·07, 1·56	1·38	1·13, 1·67
Recurrent stone former		1·13	0·96, 1·33	1·30	1·08, 1·56	1·23	1·01, 1·50	1·25	1·01, 1·56

eGFR, estimated glomerular filtration rate.

* Values were log-transformed.

† After restricting to participants with normal kidney function, the numbers of participants for total plain water/fluid intake and other hydration indicators were 3103, 3491 and 3977, respectively.

Model 1: univariate analysis.

Model 2: further adjusted for age, sex, race/ethnicity and education levels.

Model 3: further adjusted for BMI, smoking, alcohol, hypertension, diabetes mellitus, eGFR and diuretics use.

Model 4: further restricted to participants with normal kidney function (eGFR≥ 60 ml/min/1·73 m^2^).

Current guidelines include general recommendations regarding fluid consumption for preventing recurrence nephrolithiasis, for example, maintaining a daily fluid intake of 2·5 or 3 l and a daily urine output of 2·0 or 2·5 l^(9,33)^. These general recommendations were associated with a lower risk of overall nephrolithiasis but not recurrent nephrolithiasis (Table 4).

**Table 4:** OR (95 % CI) of nephrolithiasis by commonly recommended hydration practices. Reference group comprises stone-free participants

Common hydration practice	History of kidney stone		First kidney stone*		Recurrent kidney stone*	
	OR	95 % CI	OR	95 % CI	OR	95 % CI
Water intake						
Daily water intake >2500 ml/d	0·52	0·31, 0·85	0·47	0·24, 0·90	0·59	0·27, 1·30
Daily water intake >3000 ml/d	0·47	0·24, 0·93	0·54	0·23, 1·25	0·35	0·10, 1·19
Total fluid intake > 5000 g/d	0·57	0·35, 0·93	0·58	0·28, 1·19	0·54	0·28, 1·02
Urine flow rate > 2 l/d	0·66	0·49, 0·89	0·67	0·46, 0·97	0·65	0·40, 1·05
Urine flow rate > 2·5 l/d	0·63	0·41, 0·98	0·66	0·39, 1·11	0·58	0·26, 1·28

* Analyses were performed using the full-adjusted multinomial logistic regression model.

## Discussion

In this large cross-sectional study based on the NHANES 2009–2012 cycles, we observed that better hydration status (indirectly measured using urine concentration markers, blood osmolality and renal response to altered body hydration) was significantly associated with lower nephrolithiasis risk, and high urine output and FWC were significantly associated with low recurrent nephrolithiasis risk. Our study verified that maintaining a daily water intake of >2·5 l or daily urine output of >2 l can help prevent the first occurrence of kidney stone formation.

The Institute of Medicine of the National Academy of Sciences, in 2005, had attempted to improve daily water intake recommendations with the publication of the updated Dietary Reference Intake recommendations^(34)^. However, the estimated average requirement for water cannot be determined due to interindividual variation in basal metabolism and external environment; therefore, an ‘adequate intake (AI)’ indicator was proposed. In 2013, a systematic review of clinical trials suggested that increased fluid intake halved recurrent composite stone risk compared with no treatment despite the low-strength evidence^(35)^. For incident kidney stone, until recently two studies from UK biobanks supported the high intake of fluid, particularly coffee and tea, were associated with a reduction in kidney stones^(36,37)^. However, no association with decreased risk of hospitalisation for a first kidney stone was observed for increased water intake^(36)^. By contrast, few studies have investigated the role of hydration status in primary renal stone prevention; hence, the results of the present study represent the first step towards a comprehensive evaluation of hydration status and the prevalence of nephrolithiasis in general adult populations^(34)^.

Encouraging water and fluid intake to maintain adequate urine output is common advice that urology clinics communicate to stone formers. This cross-sectional study, however, revealed poor adherence to water and fluid intake advice among self-reporting stone formers, who reported diets characterised by lower fluid intake and showed a state of relative dehydration, likely indicated that despite a previous stone the participants continued with lower levels of hydration compared to other participants. A previous study has also highlighted the problem of non-adherence among stone formers who used prophylactic medication^(38)^. Our findings may inform efforts to redesign current counselling models for tracking and monitoring patient adherence, for example, mobile technology can be integrated into patient education programmes to monitor patients’ urine colour and remind them to adjust their water intake accordingly^(39)^.

Although our study indicated that better hydration (measured by blood osmolality, U_osm_:B_osm_ and FWC) was associated with a lower risk of kidney stone formation, the regular use of these specific hydration indicators is not clinically practical because blood sampling is required. By contrast, urine osmolality is clinically useful because it is non-invasive and less affected by sociodemographic factors (i.e. less variation in results) compared with the more commonly used urine creatinine test^(27)^. However, whether a single measurement of spot or 24-h urine osmolality could represent longitudinal hydration status remains to be explored. Nonetheless, a recent study supported the predictive role of urine osmolality in responding to water restriction and rehydration intervention^(40)^. Further research efforts are warranted to investigate the longitudinal trajectory of hydration indicators in stone formers that would advance our understanding of intra-individual trends of water homoeostasis in kidney stone formation and how co-morbidities, such as CVD and diabetes, modify long-term hydration status^(3,4,41,42)^.

The present study had several limitations. Its cross-sectional design prevented us from evaluating temporality and controlling for unexamined confounding factors (e.g. participants’ physical activity level, geographical climate, occupation and long-term dietary habits). Differential reporting bias between stone-free participants and stone formers with respect to daily water and fluid intake might have led to biased risk assessments, although it is possible that those with previous stones might have reported higher fluid intake due to desirability bias. However, the consistent findings for self-reported daily water and fluid intake and hydration status indicator results support the robustness of our inferences. UFR was not measured using the standard 24-h method used in the NHANES 2009–2012 cycles; in the present study, the average urine collection time was 2·9 h, which might have increased the risk of measurement errors in our 24-h urine output estimations. Nevertheless, such measurement errors would have been non-differential because stone-free participants and stone formers reported similar total urine collection times. Another limitation is that the measures of hydration status (e.g. blood and urine osmolality) reflect recent levels of hydration, not necessarily long-term hydration status, although they might be correlated. Lastly, although the sensitivity and specificity of self-reported history on symptomatic nephrolithiasis were adequate^(43)^, the prevalence of nephrolithiasis might have been underestimated due to the lack of image diagnoses. Similarly, the outcome of recurrent nephrolithiasis was also collected through an interview format, and therefore a limitation of recall bias may diminish the internal validity of the present study.

## Conclusion

Our findings support that current guidelines’ recommendations for fluid intake can be effective in both primary and secondary nephrolithiasis prevention, if they were implemented. Maintaining appropriate hydration and urine output levels are essential for preventing recurrent stone formation. These findings support the evaluation of plain water intake, which is easier for patients to understand and monitor, as a tool for nephrolithiasis prevention. However, our study also indicates that non-adherence to simple water and fluid intake recommendations remains a primary barrier among stone formers. The development of novel interventions to improve long-term lifestyle adherence should be a patient care priority.

## Supplementary material

For supplementary material accompanying this paper visit https://doi.org/10.1017/S1368980022001033. click here to view supplementary material
