# Supplementary material for: Association of water intake and hydration status with risk of kidney stone formation based on NHANES 2009–2012 cycles
Source: Public Health Nutr. 2022 May 6;25(9):2403–14. doi: 10.1017/S1368980022001033 (PMC9991749; doi:10.1017/S1368980022001033)
Supplement: Supplementary file 1 [file S1368980022001033sup.zip › S1368980022001033sup002.docx]

**Supplementary Online Content**

**Supplementary Figure 1.** The medians and interquartile ranges (IQR) of the daily water and fluid intake by socio-demographic characteristics and comorbidities from participants enrolled in NHANES cycle 2009-2010 and 2011-2012.

**Supplementary Figure 2.** The medians and interquartile ranges (IQR) of urine concentration indicators by socio-demographic characteristics and comorbidities from participants enrolled in NHANES cycle 2009-2010 and 2011-2012.

**Supplementary Figure 3.** The medians and interquartile ranges (IQR) of blood-osmolality based hydration indicators by socio-demographic characteristics and comorbidities from participants enrolled in NHANES cycle 2009-2010 and 2011-2012.

**Supplementary Figure 4.** Correlation matrix of Spearman correlation coefficients among analyzed factors related to kidney stones.

**Supplementary figure 1.** The medians and interquartile ranges (IQR) of the daily water and fluid intake by socio-demographic characteristics and comorbidities from participants enrolled in NHANES cycle 2009-2010 and 2011-2012.

**
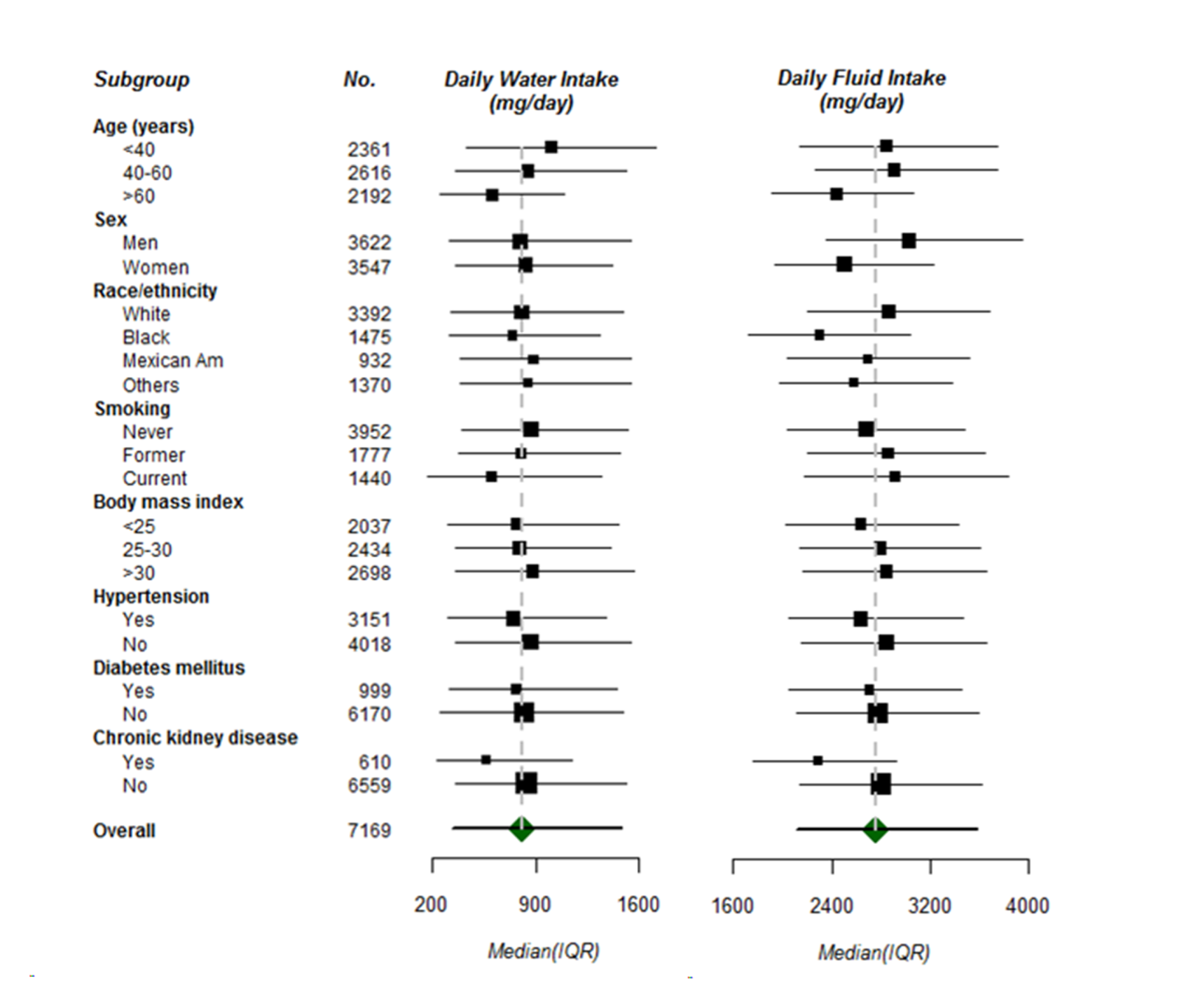
**

**Supplementary figure 2.** The medians and interquartile ranges (IQR) of urine concentration indicators by socio-demographic characteristics and comorbidities from participants enrolled in NHANES cycle 2009-2010 and 2011-2012.


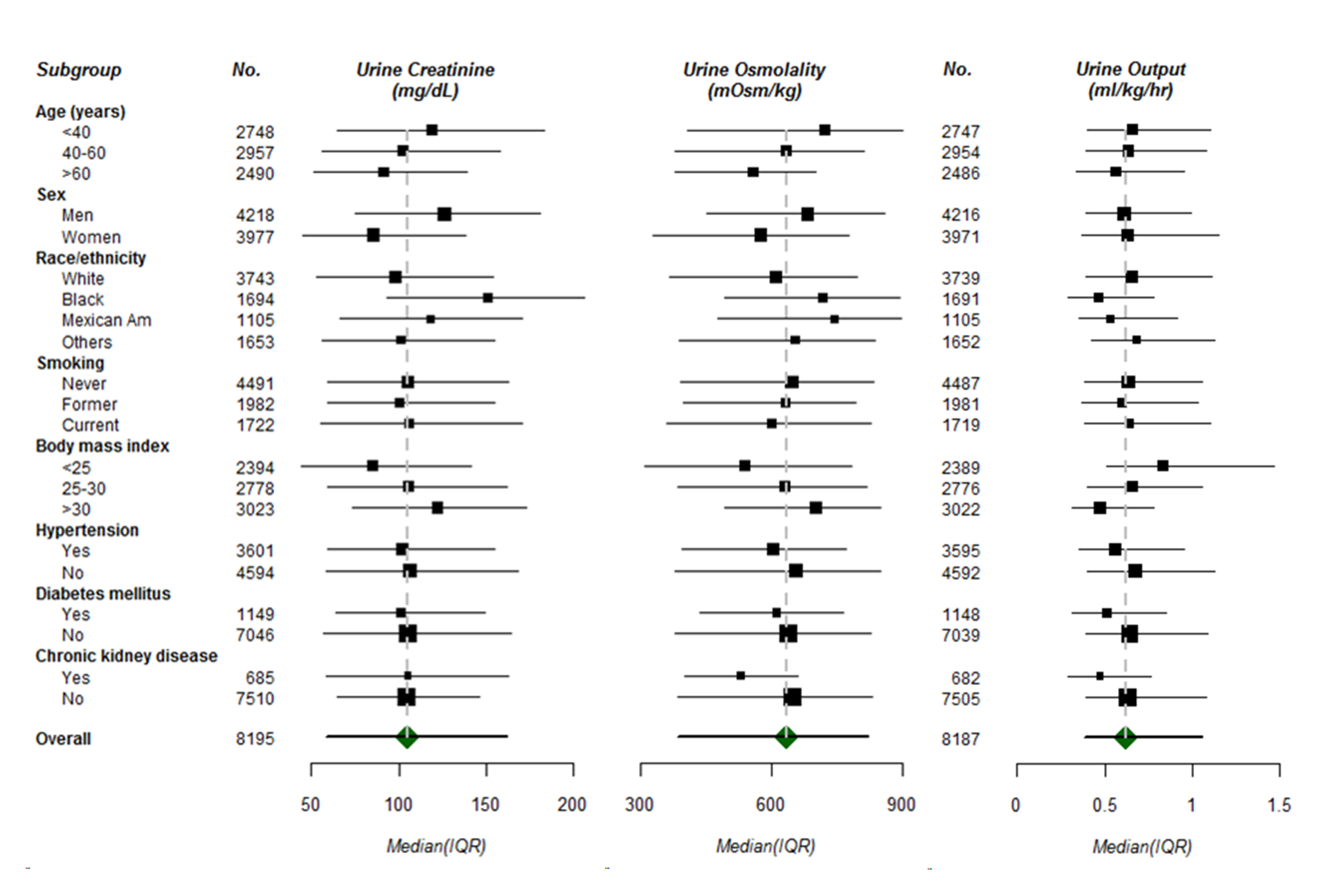


**Supplementary figure 3.** The medians and interquartile ranges (IQR) of blood-osmolality based hydration indicators by socio-demographic characteristics and comorbidities from participants enrolled in NHANES cycle 2009-2010 and 2011-2012.

**
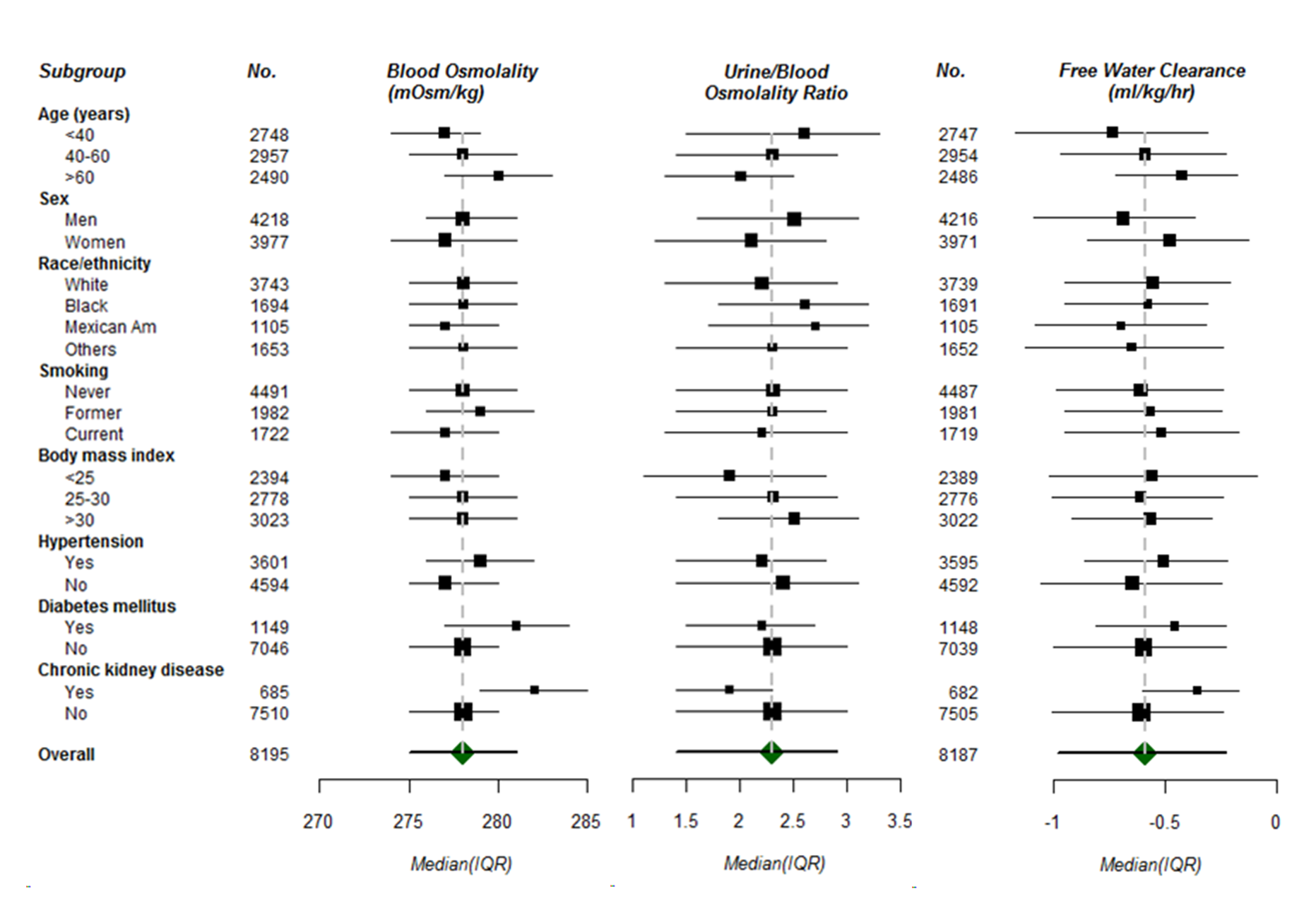
**

**Supplementary Figure 4.** Correlation matrix of Spearman correlation coefficients among analyzed factors related to kidney stones. U-to-B Osmolality ratio, urine-to-blood osmolality ratio; FWC, free water clearance; UFR, urine flow rate; TFI, total fluid intake; TPWI, total plain water intake.


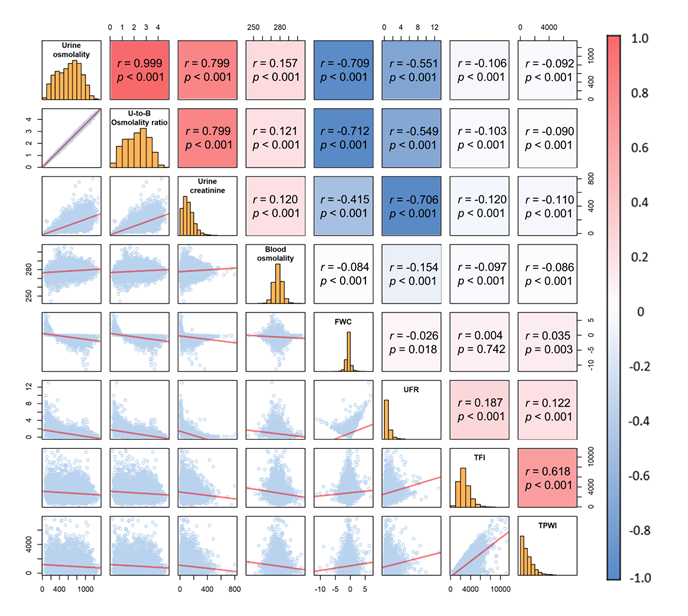


[1] Yeh HC, Lin YS, Kuo CC, Weidemann D, Weaver V, Fadrowski J, et al. Urine osmolality in the US population: implications for environmental biomonitoring. Environmental research. 2015;136:482-90.

[2] National Center for Health Statistics (NCHS). National Health and Nutrition Examination Survey Data. 2011 - 2012 Data Documentation, Codebook, and Frequencies: Prescription Medications (RXQ_RX_G). In: U.S. Department of Health and Human Services CfDCaP, editor. Hyattsville, MD2012.
